# Supplementary material for: Nervous Necrosis Virus Modulation of European Sea Bass (Dicentrarchus labrax, L.) Immune Genes and Transcriptome towards Establishment of Virus Carrier State
Source: Int J Mol Sci. 2023 Nov 22;24(23):16613. doi: 10.3390/ijms242316613 (PMC10706053; doi:10.3390/ijms242316613)
Supplement: Supplementary file 1 [file ijms-24-16613-s001.zip › ijms-2704659-supplementary.pdf]

International Journal of
Molecular Sciences

MDPI

Article

Nervous Necrosis Virus Modulation of European Sea Bass (*Dicentrarchus labrax*, L.) Immune Genes and Transcriptome towards Establishment of Virus Carrier State

Dimitra K. Toubanaki [1], Antonia Efstathiou [1], Odysseas-Panagiotis Tzortzatos [1], Michail-Aggelos Valsamidis [2], Leonidas Papaharisis [3], Vasileios Bakopoulos [2] and Evdokia Karagouni [1,*]

Supplementary material

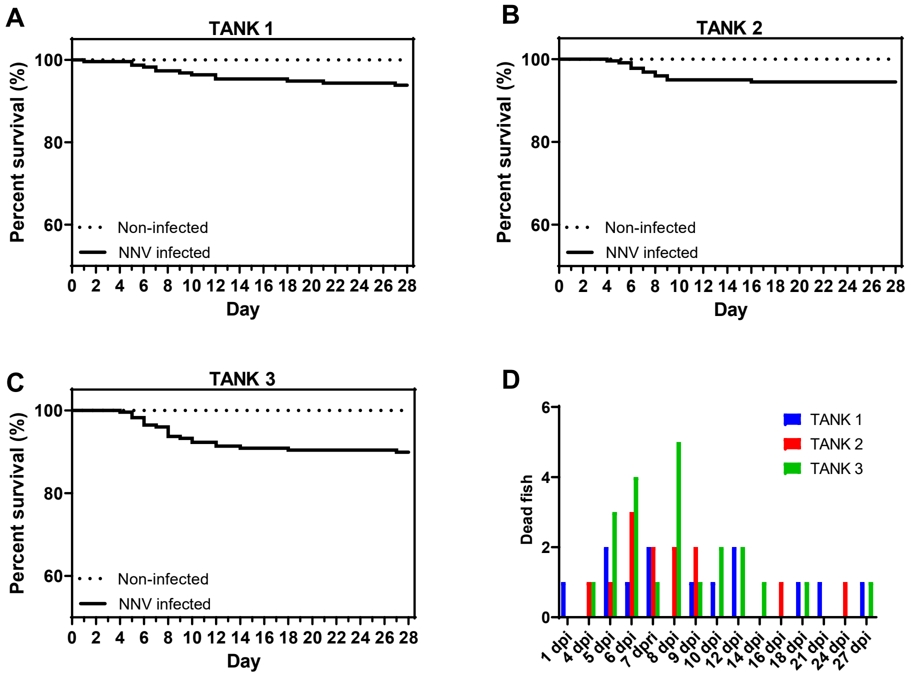

Figure S1. Cumulative survival of NNV challenged (continuous line) and non-infected (dashed line) fish groups in tank 1 (A), tank 2 (B) and tank 3 (C). (D) Dead fish in each experimental tank. Two-way ANOVA with Tukey's post test was applied to determine differences between dead fish number in each tank at different sampling time points. No significant differences were determined.

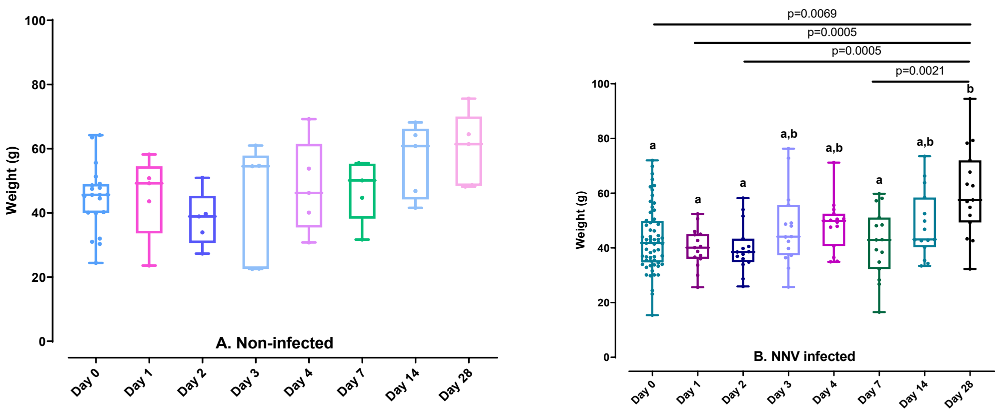

Figure S2. Weights of **(A)** non-infected and **(B)** NNV-challenged European sea bass for 0 to 28 dpi time points. Data are presented as bounds of box and whisker plots with min-to-max values. The line represents the median (non-infected: n = 5 (20 for 0 dpi); NNV infected: n = 15 (60 for 0 dpi)), the whiskers show the data range and the box shows the interquartile range. The unpaired t-test was used to determine statistical differences between infected and non-infected groups and one-way ANOVA with Dunn's post hoc test was applied to determine differences between sampling time points. Different lower-case letters denote statistically significant differences.

The condition factor (K) of a fish reflects physical and biological circumstances and fluctuations by interaction among feeding conditions, parasitic infections and physiological factors. This also indicates the changes in food reserves and therefore an indicator of the general fish condition [1].

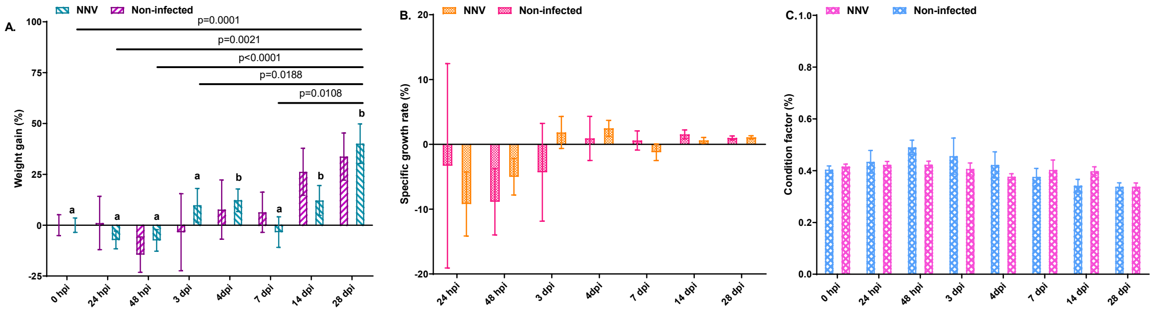

Figure S3. **(A)** Weight gain, **(B)** specific growth rate and **(C)** condition factor, of non-infected and NNV-challenged European sea bass for 0 to 28 dpi time points. Data are presented as means ± SEM (non-infected: n = 5 (20 for 0 dpi); NNV infected: n = 15 (60 for 0 dpi)). Statistical differences were determined by two-way ANOVA followed by Tukey's multiple comparison test. Different lower-case letters denote statistically significant differences.

Splenosomatic index is the weight of the spleen expressed as a percentage of total body weight. Alterations in this index could indicate an abnormal condition in the spleen such as necrosis or swelling due to infection [2].

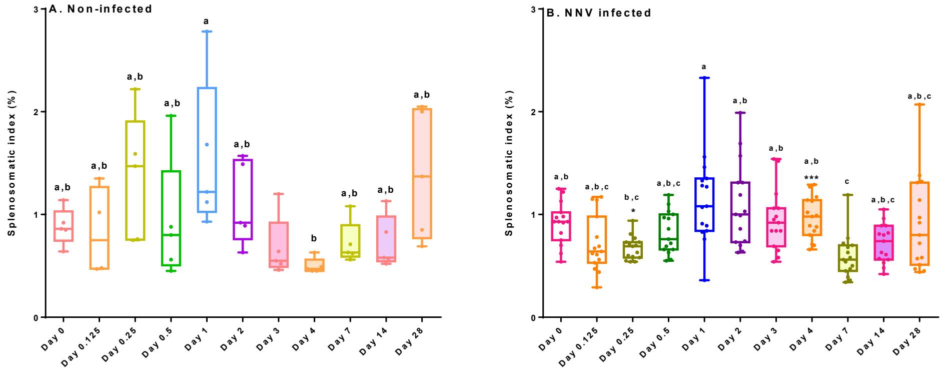

Figure S4. Splenosomatic indexes of **(A)** non-infected and **(B)** NNV-challenged European sea bass for 0 to 28 dpi time points. Data are presented as bounds of box and whisker plots with min-to-max values. The line represents the median (non-infected: n = 5; NNV challenged: n = 15), the whiskers show the data range and the box shows the interquartile range. The unpaired t-test was used to determine statistical differences between infected and non-infected groups (one asterisk indicate p< 0.05, three asterisks indicate p< 0.001). One-way ANOVA with Dunn's post hoc test were applied to determine differences between sampling time points. Different lower-case letters denote statistically significant differences.

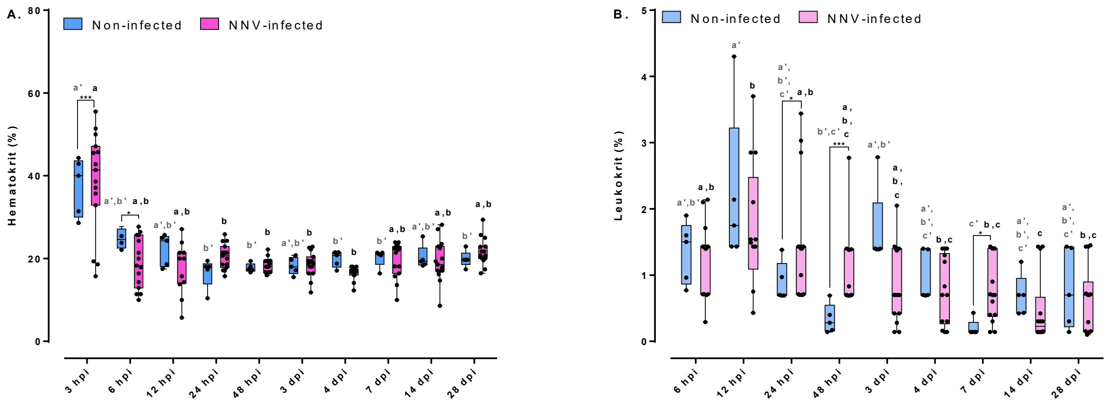

Figure S5. (A) Hematockrit (Hct) and **(B)** leukokrit (Lct) of non-infected and NNV-challenged European sea bass for 0 to 28 dpi time points. Data are presented as bounds of box and whisker plots with min-to-max values. The line represents the median (non-infected: n = 5; NNV challenged: n = 15), the whiskers show the data range and the box shows the interquartile range. The unpaired t-test was used to determine statistical differences between infected and non-infected groups (one asterisk indicate p< 0.05, three asterisks indicate p< 0.001). One-way ANOVA with Dunn's post hoc test were applied to determine differences between sampling time points. Different lower-case letters denote statistically significant differences.

Table S1. Summary of the analyzed RNA-seq libraries total reads and alignment statistics.

Sample	Total reads	Aligned one time	Aligned multiple times	Overall Alignment	Not Aligned
Inf 14dpi_1	24,860,558	3,590,113 (14.4%)	19,079,522 (76.7%)	22,669,635 (91.2%)	2,190,923 (8.8%)
Inf 14dpi_2	23,822,187	3,463,233 (14.5%)	18,883,599 (79.3%)	22,346,832 (93.8%)	1,475,355 (6.2%)
Inf 14dpi_3	28,617,650	5,471,226 (19.1%)	20,071,299 (70.1%)	25,542,525 (89.2%)	3,075,125 (10.7%)
Inf 14dpi_4	14,400,548	1,875,214 (13%)	10,904,782 (75.7%)	12,779,996 (88.8%)	1,620,552 (11.2%)
Inf 14dpi_5	15,439,357	1,974,009 (12.8%)	12,299,717 (79.7%)	14,273,726 (92.4%)	1,165,631 (7.5%)
Non Inf 14dpi_1	13,151,000	1,949,129 (14.8%)	10,062,151 (76.5%)	12,011,280 (91.3%)	1,139,720 (8.7%)
Non Inf 14dpi_2	17,669,423	2,776,674 (15.7%)	13,516,718 (76.5%)	16,293,392 (92.2%)	1,376,031 (7.8%)
Non Inf 14dpi_3	15,990,321	2,222,783 (13.9%)	12,727,551 (79.6%)	14,950,334 (93.5%)	1,039,987 (6.5%)
Non Inf 14dpi_4	15,007,899	2,143,027 (14.8%)	12,295,366 (81.9%)	14,438,393 (96.2%)	569,506 (3.8%)

Table S2. Primers and probes used in the present work.

Gene	Primer sequence (5′ - 3′)	Accession number	Product size (bp)	Efficiency (%)	Reference
NNV load quantification					
**Primers**	oPVP154: TCCAAGCCGGTCCTAGTCAA	N/A	168/171	94	[3]
	oPVP155: CACGAACGTKCGCATCTCGT				
**Taqman probe**	tqPVP16: Cy5-CGATCGATCAGCAC-CTSGTCBHQ2	N/A	N/A	N/A	[3]
Interferon pathway					
**IRF7**	Up: GGTGGTTCCTCTGATCTGTCG	KP861885.1	89	99	Present work
	Dp: TCTGTAGGCTGACATTGGCG				
**ISG12**	Up: AACATGGACCCTGGTACAGC	FN665389.1	141	102	Present work
	Dp: CGCAGCAACAGACATCACTT				
**MxA**	Up: AAAGGGACAGCCAGAGAACA	JN807551.1	97	108	Present work
	Dp: ACAAGGAACAACCACCAAGC				
Cytokines					
**IL-1b**	Up: ATTACCCACCACCCACTGAC	AJ269472.1	151	97	Present work
	Dp: TCTCTTCCACTATGCTCTCCAG				
**IL-10**	Up: CTGTCCATCTTGGTCGTCTTATC	AM268529.1	119	109	Present work
	Dp: TCTGCTCTGAGTTGCCTTAAC				
**STAT3**	Up: GGTGTGGTTGGACAACATTATT	JF810898.1	100	90	Present work
	Dp: CCTCTCCCTCTCCTTACTGATA				
**TNFa**	Up: TCACCACAGAGCACTGGAAG	DQ070246.1	154	86	Present work
	Dp: GACCGATCTCCACATCACCT				
Immunoglobulins					
**IgHM**	Up: GCTGCCTCCAATAGAACATACT	KY173353.1	101	98	Present work
	Dp: CATCAACAAGCCAAGACACAAA				
T-cell markers					
**CD4**	Up: TGAACTGCGTTATGACCCTAAA	AM849811.1	82	89	Present work
	Dp: GTCTCCACTGTCTCCATCTTTC				
**CD8a**	Up: GCCTTCTTCTTCTACTCCTCATC	AJ846849.2	132	89	Present work
	Dp: CGTGTCTGTGTGTCATCATTTG				
Antimicrobials peptides					

**Hepcidin**	Up: ATCGTGGAAGATGCCGTATAAC<br>Dp: CCCTCATATTAGGACAGCAACC	KJ890399.1	92	88	Present work
Reference					
**b-actin**	Up: GATCTGGCATCACACCTTCTAC<br>Dp: TCTTCTCCCTGTTGGCTTTG	AJ537421.1	104	112	Present work

* N/A: Non applicable.

References

1. Datta, S.N.; Kaur, V.I.; Dhawan, A.; Jassal, G. Estimation of length-weight relationship and condition factor of spotted snakehead *Channa punctata* (Bloch) under different feeding regimes. Springerplus. 2013, 2:436. doi: 10.1186/2193-1801-2-436.

2. Gupta, N.; Gupta, D.K.; Sharma, P.K. Condition factor and organosomatic indices of parasitized *Rattus rattus* as indicators of host health. J. Parasit. Dis. 2017, 41(1):21-28. doi: 10.1007/s12639-015-0744-3.

3. Baud, M.; Cabon, J.; Salomoni, A.; Toffan, A.; Panzarin, V.; Bigarré, L. First generic one step real-time Taqman RT-PCR targeting the RNA1 of betanodaviruses. Virol. Methods 2015, 211, 1-7. doi: 10.1016/j.jviromet.2014.09.016.

Disclaimer/Publisher's Note: The statements, opinions and data contained in all publications are solely those of the individual author(s) and contributor(s) and not of MDPI and/or the editor(s). MDPI and/or the editor(s) disclaim responsibility for any injury to people or property resulting from any ideas, methods, instructions or products referred to in the content.
